# Diverse Beliefs, Diverse Bodies: Prevalence of Body Dissatisfaction and Disordered Eating in Muslim vs. Non-Muslim Females – A Systematic Review

**DOI:** 10.1192/bjo.2025.10190

**Published:** 2025-06-20

**Authors:** Nesrin Al-Dabbas, Aliki Pouli, Katja Umla-Runge

**Affiliations:** ^1^Cardiff University, Cardiff, United Kingdom; ^2^Private sector, Amman, Jordan

## Abstract

**Aims:** Body dissatisfaction and disordered eating behaviours are significant global health concerns, yet their prevalence and predictors among Muslim females remain underexplored. This systematic review aimed to compare the prevalence and associated factors of body dissatisfaction and disordered eating behaviours in Muslim and non-Muslim females, with a focus on religiosity and cultural practices.

**Methods:** The inclusion criteria encompassed primary observational studies published in English that compared body dissatisfaction and disordered eating behaviours between Muslim and non-Muslim females. Studies that did not report prevalence data on these outcomes or included male participants were excluded. Following PRISMA guidelines, a comprehensive search was conducted across Medline, Embase, PsycINFO, and Scopus up to August 18, 2024, supplemented by references and grey literature. Methodological quality was assessed using the ROBINS-E and JBI Prevalence tools. Relevant data were extracted and analysed through quantitative and narrative syntheses.

**Results:** Eleven studies met the eligibility criteria. Overall, findings indicated lower rates of body dissatisfaction and disordered eating behaviours among Muslim females, particularly among those practising veiling, compared with non-Muslim females. However, conflicting results were noted in several studies, possibly due to acculturation and sociocultural stressors. The heterogeneity in study methodologies and cultural contexts limited the generalizability of these findings. While religiosity appeared to be a protective factor, the variability in results underscores the need for more robust, longitudinal studies to better understand the complex interplay of religious, cultural, and psychosocial factors.

**Conclusion:** This review highlights the importance of integrating cultural and religious contexts into effective interventions and prevention strategies for body dissatisfaction and disordered eating in Muslim populations.

